# Structural basis of INTAC-regulated transcription

**DOI:** 10.1093/procel/pwad010

**Published:** 2023-03-04

**Authors:** Hai Zheng, Qianwei Jin, Xinxin Wang, Yilun Qi, Weida Liu, Yulei Ren, Dan Zhao, Fei Xavier Chen, Jingdong Cheng, Xizi Chen, Yanhui Xu

**Affiliations:** Fudan University Shanghai Cancer Center, Institutes of Biomedical Sciences, State Key Laboratory of Genetic Engineering, Shanghai Key Laboratory of Radiation Oncology, and Shanghai Key Laboratory of Medical Epigenetics, Shanghai Medical College of Fudan University, Shanghai 200032, China; Fudan University Shanghai Cancer Center, Institutes of Biomedical Sciences, State Key Laboratory of Genetic Engineering, Shanghai Key Laboratory of Radiation Oncology, and Shanghai Key Laboratory of Medical Epigenetics, Shanghai Medical College of Fudan University, Shanghai 200032, China; Fudan University Shanghai Cancer Center, Institutes of Biomedical Sciences, State Key Laboratory of Genetic Engineering, Shanghai Key Laboratory of Radiation Oncology, and Shanghai Key Laboratory of Medical Epigenetics, Shanghai Medical College of Fudan University, Shanghai 200032, China; Fudan University Shanghai Cancer Center, Institutes of Biomedical Sciences, State Key Laboratory of Genetic Engineering, Shanghai Key Laboratory of Radiation Oncology, and Shanghai Key Laboratory of Medical Epigenetics, Shanghai Medical College of Fudan University, Shanghai 200032, China; Fudan University Shanghai Cancer Center, Institutes of Biomedical Sciences, State Key Laboratory of Genetic Engineering, Shanghai Key Laboratory of Radiation Oncology, and Shanghai Key Laboratory of Medical Epigenetics, Shanghai Medical College of Fudan University, Shanghai 200032, China; Fudan University Shanghai Cancer Center, Institutes of Biomedical Sciences, State Key Laboratory of Genetic Engineering, Shanghai Key Laboratory of Radiation Oncology, and Shanghai Key Laboratory of Medical Epigenetics, Shanghai Medical College of Fudan University, Shanghai 200032, China; Fudan University Shanghai Cancer Center, Institutes of Biomedical Sciences, State Key Laboratory of Genetic Engineering, Shanghai Key Laboratory of Radiation Oncology, and Shanghai Key Laboratory of Medical Epigenetics, Shanghai Medical College of Fudan University, Shanghai 200032, China; Fudan University Shanghai Cancer Center, Institutes of Biomedical Sciences, State Key Laboratory of Genetic Engineering, Shanghai Key Laboratory of Radiation Oncology, and Shanghai Key Laboratory of Medical Epigenetics, Shanghai Medical College of Fudan University, Shanghai 200032, China; Fudan University Shanghai Cancer Center, Institutes of Biomedical Sciences, State Key Laboratory of Genetic Engineering, Shanghai Key Laboratory of Radiation Oncology, and Shanghai Key Laboratory of Medical Epigenetics, Shanghai Medical College of Fudan University, Shanghai 200032, China; Fudan University Shanghai Cancer Center, Institutes of Biomedical Sciences, State Key Laboratory of Genetic Engineering, Shanghai Key Laboratory of Radiation Oncology, and Shanghai Key Laboratory of Medical Epigenetics, Shanghai Medical College of Fudan University, Shanghai 200032, China; Fudan University Shanghai Cancer Center, Institutes of Biomedical Sciences, State Key Laboratory of Genetic Engineering, Shanghai Key Laboratory of Radiation Oncology, and Shanghai Key Laboratory of Medical Epigenetics, Shanghai Medical College of Fudan University, Shanghai 200032, China; The International Co-laboratory of Medical Epigenetics and Metabolism, Ministry of Science and Technology, China, Department of Systems Biology for Medicine, School of Basic Medical Sciences, Shanghai Medical College of Fudan University, Shanghai 200032, China; Human Phenome Institute, Collaborative Innovation Center of Genetics and Development, School of Life Sciences, Fudan University, Shanghai 200433, China

**Dear Editor,**

Eukaryotic transcription by RNA polymerase II (Pol II) is a strictly regulated process that involves the interplay of numerous factors. Promoter-proximal pausing is a regulatory mechanism that connects transcription initiation and productive elongation in metazoans (Core and Adelman, 2019). Pol II forms a paused elongation complex (PEC) through binding of two transcriptional regulation factors DSIF and NELF (Vos et al., 2018). Following the duration of pausing, Pol II either proceeds into productive elongation or undergoes promoter-proximal premature transcription termination (PTT) (Kamieniarz-Gdula and Proudfoot, 2019), which plays a decisive role in determining transcriptional outputs.

In contrast to the well-characterized pause release and productive elongation, the mechanism of PTT remains largely unknown. Integrator complex functions as an RNA endonuclease to cleave different classes of RNAs (Skaar et al., 2015). More recent studies discovered that Integrator is enriched in the proximity of gene promoters and can associate with paused Pol II bound by DSIF and NELF (Stadelmayer et al., 2014; Yamamoto et al., 2014) to trigger PTT and repress gene activity (Huang et al., 2020). We have recently found that Integrator associates with protein phosphatase 2A core enzyme (PP2A-AC) and dephosphorylates the C-terminal domain (CTD) of Pol II and determined the structure of Integrator-containing PP2A-AC (termed INTAC), showing how the RNA endonuclease and protein phosphatase are organized in the INTAC complex (Zheng et al., 2020). Despite these studies, it remains elusive how INTAC, especially its two catalytic modules, is structurally organized and functionally coordinated in the context of PEC and how INTAC works with PEC in PTT.

To investigate the mechanistic implications of INTAC in promoter-proximal pausing, the INTAC-PEC complex was assembled using purified human INTAC (Zheng et al., 2020), NELF, DSIF, *S. scrofa* Pol II (Chen et al., 2021), and a DNA–RNA scaffold (Fig. S1A and S1B). The assembled INTAC-PEC complex was subjected to gradient fixation (GraFix), followed by cryo-electron microscopy (cryo-EM) single-particle reconstruction (Fig. S2). The cryo-EM map was refined to 4.2 Å resolution and the maps of subcomplexes were improved to near-atomic (3.5–3.8 Å) resolution by focused refinement. Structural model was built by fitting previously determined structures of INTAC (Zheng et al., 2020) and PEC (Vos et al., 2018) into the cryo-EM maps followed by manual adjustment (Table S1). Structural analysis of INTAC-PEC contacts was largely based on previously reported high-resolution structures docked into the overall and locally masked cryo-EM maps.

The INTAC-PEC complex structure reveals a compact fold with approximate dimensions of ~300 × 270 × 260 Å^3^ (Figs. 1A, 1B and S4, Video S1). Structural comparison shows that the phosphatase and endonuclease modules of INTAC are more separated in INTAC-PEC to fit the binding of PEC. A previously undetected tail module extends out of the bottom of the backbone module and folds back to bind Pol II (Fig. S4). The PEC complex sits above the main body of INTAC through interface-I/-II/-V and is further stabilized by two INTAC protrusions on opposite sides at interface-III/-IV.

**Figure 1.**
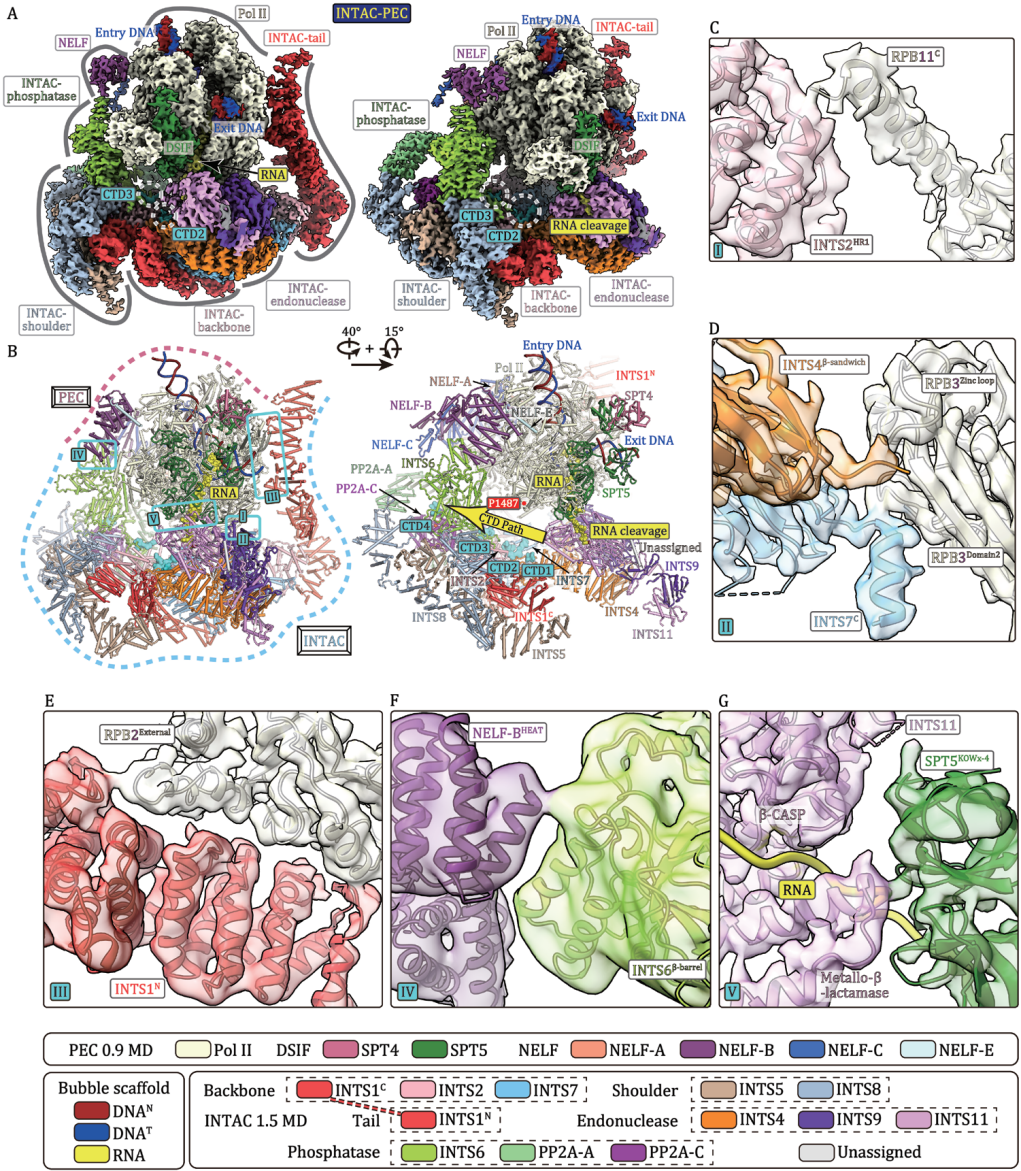
**Structure of INTAC-PEC complex and interactions between INTAC and PEC.** (A and B) Composite cryo-EM map (A) and structural model (B) of INTAC-PEC shown in two different views. Five INTAC-PEC interfaces are indicated and four putative Pol II CTD fragments are shown in surface representation. The same color scheme was used in all of the figures if not otherwise specified. Putative CTD-binding path on INTAC and RNA cleavage site in INTS11 are highlighted. (C–G) Close-up views of the interactions between INTAC and PEC with cryo-EM maps shown in transparent surface and structural models shown in cartoon.

INTAC makes three direct contacts with Pol II (Figs. 1C–E and S5). At the interface-I, the C-terminal α-helix of RPB11 contacts the helical repeat 1 of INTS2 (INTS2^HR1^) (Zheng et al., 2020). At the interface-II, the C-terminal helix of INTS7 binds the domain2 of RPB3 and the C-terminal end of INTS4 bridges the contact between INTS9 and the zinc loop of RPB3. The N-terminal HEAT (huntingtin, elongation factor 3, protein phosphatase 2A, and TOR1) domain of INTS1 adopts an arch-shaped fold and forms a tail module. The two RPB2 external domains of Pol II contact the tail module on the convex ridge at the interface-III, suggesting a PEC-mediated tail stabilization and a potential function of tail in recruitment of PEC.

INTS6 bridges the phosphatase of INTAC and the NELF-B-NELF-E lobe of PEC (Figs. 1F and S5). At the interface-IV, the exposed end of the INTS6 β-barrel domain contacts the HEAT domain of NELF-B, consistent with the known interaction between Integrator and NELF (Stadelmayer et al., 2014; Yamamoto et al., 2014). At the interface-V, the SPT5 KOWx-4 domain packs on Pol II, stabilizes the exit RNA, and contacts the INTS11 (Figs. 1G and S5). As discussed below, the interaction brings RNA to INTS11 for cleavage.

The human Pol II CTD consists of 52 consensus heptapeptide repeats (Tyr1-Ser2-Pro3-Thr4-Ser5-Pro6-Ser7) and the phosphorylation levels at Ser2, Ser5, and Ser7 change dynamically throughout the transcription cycle (Harlen and Churchman, 2017). Cryo-EM map reveals four putative Pol II CTD segments, indicative of a potential CTD-binding path toward the active center of PP2A-C for dephosphorylation (Figs. 2A and S6, Video S2). Pol II and INTAC generate a center-hollowed cradle with the CTD-binding path of INTAC being ~50 Å away from the last modeled RPB1 residue (P1487).

**Figure 2.**
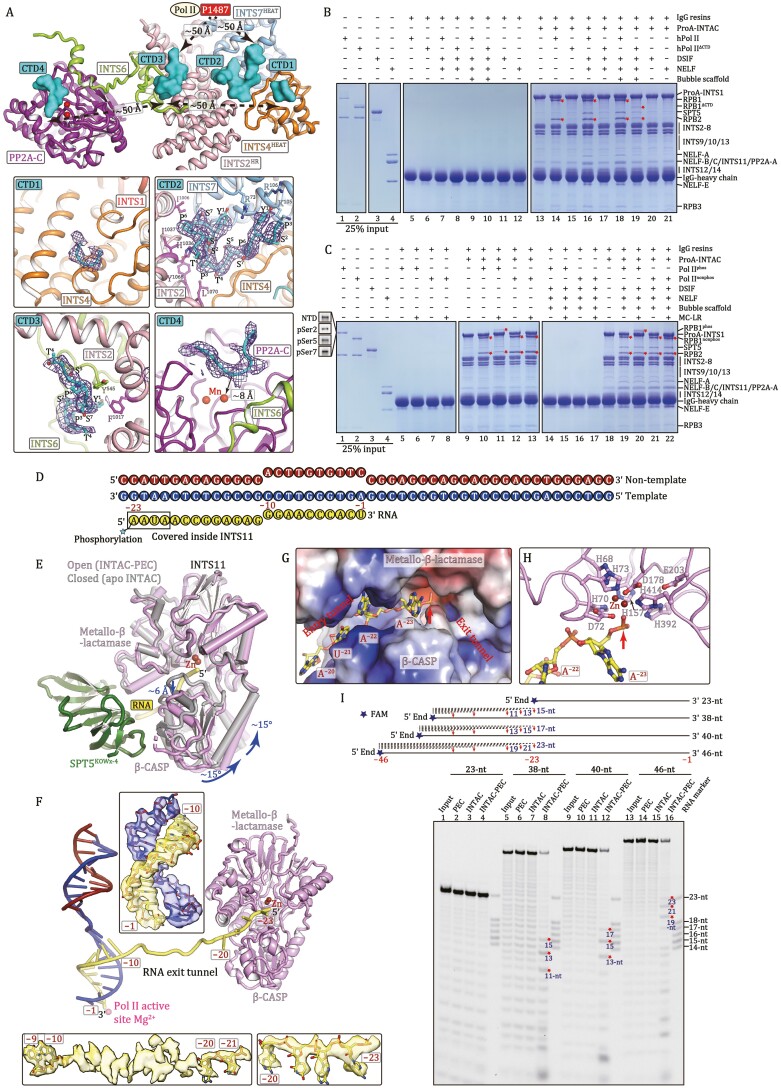
**Pol II CTD is required for INTAC-Pol II interaction and RNA is recognized and cleaved by INTS11 in an active conformation.** (A) Close-up view of Pol II CTD-binding sites on INTAC. Four putative CTD fragments are shown in surface and non-relevant subunits/domains were omitted for simplicity. The position of residue P1487 (the last modeled residue) of RPB1 is shown to indicate its distance to CTD fragments. The bottom panels show close-up views of the interactions between CTDs and INTAC with CTDs shown in cryo-EM maps (purple meshes) and structural models. Residues involved in interactions of CTD-2 and CTD-3 with INTAC are shown in sticks. (B and C) *In vitro* pulldown assays using purified INTAC and PEC subcomplexes. Protein A (ProA)-tagged INTAC was incubated with indicated subcomplexes and immobilized onto IgG resins. The unbound proteins were washed and immobilized samples were subjected to SDS-PAGE and Coomassie blue staining. hPol II and hPol II^ΔCTD^ represent human Pol II and CTD-truncated human Pol II overexpressed in Expi293F cells, respectively, and Pol II^phos^ represents *S. scrofa* Pol II that underwent *in vitro* phosphorylation by TFIIH. RPB1^phos^ contains the phosphorylation of CTD at Ser2, Ser5, and Ser7 evaluated by Western blotting, highlighted in (C). Red stars indicate the positions of RPB1 (phos and nonphos) and RPB2, reflecting the binding of Pol II in the reactions. (D) Schematic diagram of nucleic acid scaffold used for structural analysis. Template DNA is colored in blue, non-template DNA is in dark red and RNA is in yellow. (E) Assembly of INTAC-PEC leads to activation of INTS11. The structures of INTS11 in the apo INTAC (gray) (Zheng et al., 2020) and INTAC-PEC (pink, this study) are superimposed. Rotation of the β-CASP domain and opening of the RNA entry tunnel are indicated with blue arrows. The movement of INTS11 likely results from binding of PEC and/or RNA. (F) Structural model of DNA–RNA scaffold. Cryo-EM maps of three parts for RNA are shown. RNA fragments between −10 and −20 were not assigned due to the weak cryo-EM density. (G and H) Recognition of RNA by INTS11 within the RNA entry tunnel (G) and above the active site (H). (G) Electrostatic potential surface of INTS11 is shown and RNA is shown in sticks. (H) Organization of the catalytic center and positioning of RNA for cleavage. (I) The partial RNA cleavage assay. Schematic diagram shows RNAs with representative products indicated in dashed lines. Note that only FAM-labeled 5ʹ end-containing products could be visualized. Representative RNA cleavage products are highlighted in red stars in the Gel. The synthesized RNAs contain trace amount short fragments, which were well separated as ladders in the Gel.

The CTD-1 to CTD-3 segments are sequentially arrayed on the surface of INTAC backbone, spanning ~50 Å in length (Figs. 2A and S6, Video S2). Weak cryo-EM map suggests a putative CTD fragment (CTD-1) packing against a relatively hydrophobic pocket of the HEAT repeat of INTS4. The CTD-2 (~14 residues) forms a U-turn coil and packs against the molecular junction of INTS2, INTS4, and INTS7 and is stabilized by a network on interactions. Particularly, two tyrosine (Y^1^) residues anchor on the surface of INTS7 HEAT repeat and sandwich residue R73 of INTS7, generating stacking interaction. The CTD-3 (~8 residues) anchors into a hydrophobic pocket formed by INTS2 and an extending loop of INTS6.

Relatively weak cryo-EM map was observed above the catalytic pocket of PP2A-C (Figs. 2A, S6C and S6G). The density is possibly derived from Pol II CTD or the N-terminal tail of INTS6 and was termed CTD-4 for simplicity. This U-shaped fragment consists of ~8 residues and is suspended above the substrate-binding groove of PP2A-C, in a manner similar to microcystin-LR (MC-LR, PP2A inhibitor) in the PP2A holoenzyme structure (Xu et al., 2006). The fragment is positioned near the catalytic manganese cation, suggesting a position of phosphorylated Ser5 residue of CTD for dephosphorylation.

To further investigate how INTAC-PEC complex is assembled, we performed an *in vitro* pulldown assay using immobilized INTAC and individually purified human Pol II (hPol II), CTD-truncated hPol II (hPol II^ΔCTD^), NELF, and DSIF (Figs. 2B and S1A). Consistent with previous studies (Zheng et al., 2020), INTAC could pull out hPol II in nearly 1:1 stoichiometry (Fig. 2B, lanes 13–14). In contrast, the deletion of CTD impaired INTAC-hPol II interaction (lane 15) and isolated CTD could pull out INTAC (Fig. S1C). The immobilized DSIF or NELF exhibited a weak but detectable binding with INTAC (Fig. S1D), consistent with previous studies showing their binding to Integrator independent of DNA or RNA (Stadelmayer et al., 2014; Yamamoto et al., 2014). In agreement with this weak interaction and their limited contact with INTAC (Fig. 1F and 1G), the addition of DSIF and NELF showed nearly undetectable effect on Pol II-INTAC interaction (Fig. 2B, lanes 14, 16). Interestingly, the addition of a DNA–RNA scaffold to hPol II^ΔCTD^, along with DSIF and NELF, caused a slight increase in binding to INTAC (lanes 17, 19), suggesting that the exiting RNA may facilitate the binding of PEC to INTAC. The above result underscores the critical role of CTD in the recruitment of INTAC to Pol II and the assembly of INTAC-PEC.

We next validate the effect of CTD phosphorylation on INTAC-Pol II interaction (Fig. 2C). The purified *S*. *scrofa* Pol II is non-phosphorylated (Pol II^nonphos^), which was subjected to *in vitro* phosphorylation by TFIIH. The reaction product (Pol II^phos^) possesses phosphorylation at Ser2, Ser5, and Ser7 sites. The immobilized INTAC bound Pol II^nonphos^ (lane 12), and dephosphorylated and bound Pol II^phos^ (lane 10). Less amount of Pol II^phos^ was pulled out (lane 11) in the presence of PP2A inhibitor, which hampered CTD dephosphorylation. In the context of PEC, INTAC showed a comparable binding to Pol II^phos^ and Pol II^nonphos^ (Fig. 2C, lanes 18–22), suggesting that the assembly of INTAC-PEC may further stabilize the binding of Pol II. The result agrees with previous study showing that Integrator binds both Pol II^nonphos^ and Pol II^phos^ (Ebmeier et al., 2017). The above structural and biochemical results suggest that multiple CTD-binding sites on INTAC may facilitate anchoring CTD repeats and recruiting nearby phosphorylated CTD to PP2A-C for persistent dephosphorylation.

Consistent with previous studies (Vos et al., 2018), the INTAC-PEC structure shows that the SPT5 KOWx-4 and KOW5 domains function as an “RNA clamp” and contact INTAC on INTS9-INTS11 heterodimer (Figs. 1A, 1B, 2E, and S7, Video S3). At the interface-V, the SPT5 KOWx-4 domain contacts the INTS11 metallo-β-lactamase domain, which bridges the RNA exit tunnel of Pol II and RNA entry tunnel of INTS11. Although DSIF, NELF, and Pol II body are not essentially required for binding to INTAC, INTAC-PEC interactions at interface-I to -V may maintain the overall modular organization and guide RNA to the active center of INTS11 for cleavage.

INTS11 exhibits a closed, inactive conformation in the structures of RNA-free INTAC (Zheng et al., 2020) and the isolated endonuclease module (INTS4-INTS9-INTS11) (Pfleiderer and Galej, 2021) (Figs. 2E and S7, Video S4). Superposition of INTS11 metallo-β-lactamase domain shows that the association of RNA-bound PEC induces a rotation of INTS11 β-CASP domain by ~15°C and an opening of the substrate-binding tunnel by ~6 Å, permitting the entry of the RNA for cleavage. Structural comparison suggests an activation of INTS11 upon assembly of INTAC-PEC.

Within the 23-nucleotide (nt) RNA used in structure determination, nucleotides − 1 to − 10 (upstream of the nucleotide addition site) form DNA–RNA hybrid within Pol II and the following strand winds out of the RNA exit tunnel and extends to INTS11, as shown in weak but noticeable cryo-EM density, consistent with its lack of protein contacts (Figs. 2D–2F and S7). Four nucleotides at the 5ʹ end insert into the RNA entry tunnel of INTS11 with the phosphate and ribose groups well-ordered. The phosphate groups face downward the RNA entry tunnel while the bases face outward and are sandwiched by the deep cleft (Fig. 2G, Video S3). The organization of catalytic pocket and the placement of RNA substrate are generally similar to that of cleavage and polyadenylation specificity factor (CPSF) CPSF73 in the histone pre-mRNA cleavage complex (HCC) (Fig. S7B), which adopts an active state, poised for the cleavage reaction (Sun et al., 2020).

We next used five different RNA substrates (23-nt, 38-nt, 40-nt, 46-nt, and 40-nt*) with fluorescence probe labeling at the 5ʹ end to perform partial RNA cleavage assay, which allowed for the detection of intermediate products (Figs. 2I, S7D and S7E). Note that 23-nt, 38-nt, 40-nt, and 46-nt substrates have the same nucleotide sequence at their equivalent regions whereas the 40-nt* RNA has different nucleotide sequences from − 40 to − 11. No RNA cleavage was observed in the reactions using PEC or INTAC. In the presence of PEC and INTAC, RNA cleavage occurred at multiple sites and the nearest site to the nucleotide addition site is nucleotide −23 (in 38-nt, 40-nt, and 46-nt substrates) or nucleotide −24 (in 40-nt* substrate) (Figs. 2I, S7D and S7E). The different cleavage patterns of the 40-nt and 40-nt* RNAs may result from slight difference in RNA conformations in the context of INTAC-PEC complex. In addition, no cleavage activity was observed for INTAC containing INTS11 catalytic mutant (E203Q) (Fig. S7D, lane 6), indicating that the observed activity indeed result from INTAC. Similar cleavage pattern was observed for RNA substrates of 38-nt, 40-nt, and 46-nt (Fig. 2I, lanes 8, 12, 16; Fig. S7D, lane 5), whereas no detectable cleavage occurred for the 23-nt RNA (Fig. 2I, lane 4). Thus, the nucleotide above INTS11 active site in the INTAC-PEC structure might be derived from nucleotide −23, because the following nucleotides (−22 to −1) would not extend to this position (Figs. 2F–2H and S7B). The above structural and biochemical results suggest that INTAC-PEC paves an RNA path from Pol II active site to the catalytic site of INTS11 to permit cleavage of nascent RNA into a product about 23-nt or 24-nt.

Our study reveals the molecular organization of INTAC-PEC and the underlying mechanism of cooperative function of INTAC and PEC in orchestrating CTD dephosphorylation and RNA cleavage for PTT. During manuscript preparation, Fianu et al. reported cryo-EM structure of Intergrator-PP2A bound to PEC (Fianu et al., 2021). The structure is generally similar to our structure except that RNA was not observed in the RNA entry tunnel of INTS11 and only one CTD fragment (CTD-2 in our study) was observed. Nevertheless, our independent study provides additional insights into RNA cleavage by INTS11 and INTAC-mediated Pol II CTD recognition and dephosphorylation.

## Supplementary Material

pwad010_suppl_Supplementary_Materials

pwad010_suppl_Supplementary_Movie_S1

pwad010_suppl_Supplementary_Movie_S2

pwad010_suppl_Supplementary_Movie_S3

pwad010_suppl_Supplementary_Movie_S4

## Footnotes

We thank Center of Cryo-Electron Microscopy at Fudan University for the supports on cryo-EM data collection and data analyses. This work was supported by grants from the National key R&D program of China (No. 2021YFA1300100), the National Natural Science Foundation of China (Nos. 32271308, 32030055, 31830107, and 31821002), the XPLORER Prize (Y.X.), the Shanghai Municipal Science and Technology Major Project (No. 2017SHZDZX01), Shanghai Municipal Science and Technology Commission (No. 19JC1411500), and the Science and Technology Major Project of Inner Mongolia Autonomous Region of China to the State Key Laboratory of Reproductive Regulation and Breeding of Grassland Livestock (No. 2020ZD0008).

H. Z. prepared the samples for structural and biochemical analyses with help from F. X. C., X. W., D. Z., and X. C; Q. J. collected the data and performed EM analyses and model building with help from Y. Q., W. L., Y. R. and J. C.; Y. X. and H. Z. wrote the manuscript; Y. X. supervised the project.

The cryo-EM map of INTAC-PEC has been deposited in the Electron Microscopy Data Bank (EMDB) with the accession code EMD-33741 and the model coordinate of INTAC-PEC has been deposited in the Protein Data Bank (PDB) with the accession code 7YCX.

Authors declare no competing interests.
