# Supplementary material for: Structural basis of INTAC-regulated transcription
Source: Protein Cell. 2023 Mar 4;14(9):698–702. doi: 10.1093/procel/pwad010 (PMC10501182; doi:10.1093/procel/pwad010)
Supplement: pwad010_suppl_Supplementary_Materials [file pwad010_suppl_supplementary_materials.pdf]

Supplemental materials

Materials and methods

Protein expression and purification

INTAC was overexpressed and purified as previously described (Zheng et al., 2020). INTAC containing the catalytic mutant INTS11 (E203Q) was purified with minor modifications. For INTAC (E203Q), only INTS11 was tagged with an N-terminal 4×Protein A to avoid the contamination of endogenous INTS11. Pol II was isolated from *S. scrofa* thymus and purified following the reported protocol (Chen et al., 2021). Four residue substitutions (G882S of RPB2, T75I of RPB3, S140N of RPB3, and S126T of RPB6) exist between *S. scrofa* and *H. sapiens* Pol II.

All the purification steps were performed at 4 °C unless otherwise stated. The two full-length open reading frames (ORFs) of human DSIF subunits (SPT4 and SPT5) were separately subcloned into a modified pCAG vector and SPT4 was tagged with an N-terminal 2×Protein A. Both plasmids were co-transfected to Expi293F cells using PEI (Polysciences) when the cells reached a density of 2.5 × 10^6/ml. After being cultured at 37 °C for 60 hours, cells were harvested and lysed in lysis buffer containing 50 mM Na-HEPES pH 7.4, 300 mM NaCl, 0.25% (w/v) CHAPS, 5 mM $MgCl_2$, 5 mM adenosine triphosphate (ATP), 10% (v/v) glycerol, 2 mM dithiothreitol (DTT), 1 mM phenylmethylsulfonyl fluoride (PMSF), 1 µg/ml aprotinin, 1 µg/ml pepstatin, 1 µg/ml leupeptin for 30 min. The lysate was clarified by centrifugation at 16,000 rotations per minute (rpm) for 30 min with JLA-16.250 rotor (Beckman Coulter), and the supernatant was incubated with immunoglobulin G (IgG) resins (Smart-Lifesciences) overnight. The resins were washed with buffer containing 30 mM Na-HEPES pH 7.4, 300 mM NaCl, 0.1% (w/v) CHAPS, 2 mM $MgCl_2$, 10% (v/v) glycerol, 2 mM DTT. After on-column cleavage by 3C protease for 4 hours, the immobilized proteins were eluted and further purified by ion exchange chromatography (Mono Q 5/5, GE Healthcare). Peak fractions were assessed by SDS-PAGE followed by Coomassie blue staining. Protein concentration was determined by measuring absorption at 280 nm and using the predicted extinction coefficient for DSIF. Pure fractions were pooled, aliquoted, snap frozen and stored at −80 °C.

NELF was prepared essentially in a similar way as described in DSIF. The four full-length ORFs of human NELF subunits (NELF-A, -B, -C, -E) were separately subcloned into a modified pCAG vector and NELF-E was tagged with an N-terminal 2×Protein A. The plasmids were co-transfected into Expi293F cells for overexpression. The cells were collected by centrifugation and resuspended in lysis buffer containing 50 mM Na-HEPES pH 7.4, 300 mM NaCl, 0.25% (w/v) CHAPS, 5 mM $MgCl_2$, 5 mM ATP, 10% (v/v) glycerol, 2 mM DTT, 1 mM PMSF, 1 µg/ml aprotinin, 1 µg/ml pepstatin, 1 µg/ml leupeptin. After cell lysis, the lysate was cleared by centrifugation and the supernatant was incubated with IgG resins (Smart-Lifesciences) for 4 hours followed by on-column digestion by 3C protease for 4 hours. The

eluate was further purified by ion exchange chromatography (Mono Q 5/5, GE Healthcare). Peak fractions were pooled and protein purity was assessed by SDS-PAGE and Coomassie blue staining. Pure NELF was concentrated and subjected to *in vitro* dephosphorylation overnight by Lambda Protein Phosphatase (Lambda PP, Beyotime Biotechnology). The dephosphorylated NELF was applied onto a Superdex200 10/300 GL column (GE Healthcare) in a buffer containing 30 mM K-HEPES pH 7.4, 150 mM KCl, 5% (v/v) glycerol, 2 mM DTT. Peak fractions containing NELF were pooled, aliquoted, snap frozen, and stored at −80 °C.

Cryo-EM sample preparation

DNA oligos were purchased from Generay Biotechnology and RNA oligos were purchased from Bioneer. All oligos were resuspended in RNase-free water (200 µM) and stored at −30 °C. The Pol II elongation complex (EC) was assembled on a bubble scaffold with the following nucleic acid sequences as previously reported with minor modifications (Vos et al., 2018a): template DNA 5′-GCT CCC AGC TCC CTG CTG GCT CCG AGT GGG TTC CGC CGC TCT CAA TGG-3′, non-template DNA 5′-CCA TTG AGA GCG GCA CTT GTG TTC CGG AGC CAG CAG GGA GCT GGG AGC-3′, and RNA 5′-Phosphorylation-AAU AAC CGG AGA GGG AAC CCA CU-3′. The scaffold contains 10 bp DNA−RNA hybrid, 10-nucleotide bubble, 13 nucleotides of exit RNA, 24 nucleotides of entry DNA and 14 nucleotides of exit DNA. To obtain the DNA−RNA hybrid, template DNA and RNA were mixed with a molar ratio of 1:1.3 and were annealed by incubating the nucleic acids at 95 °C for 10 min and then decreasing the temperature by 1 °C min^{-1} steps to a final temperature of 4 °C in a thermocycler in a buffer containing 20 mM K-HEPES pH 7.4, 60 mM KCl, 3 mM $MgCl_2$, and 5% (v/v) glycerol. All concentrations below refer to the final concentrations used in complex assembly. To assemble EC, the purified *S. scrofa* Pol II (275 pmol) was incubated with twofold molar excess of the DNA−RNA hybrid for 15 min at 30 °C, shaking at 300 rpm, followed by the addition of twofold molar excess of non-template DNA and further incubation for 15 min at 30 °C. The purified DSIF and NELF were added in a twofold molar excess relative to Pol II for the PEC reconstitution. The sample was incubated for 1 hour at 4 °C, followed by the addition of the purified INTAC (230 pmol) and incubation for another 2 hours at 4 °C. The resulting sample was subjected to gradient fixation (GraFix) (Kastner et al., 2008). The glycerol gradient was prepared using light buffer containing 8% (v/v) glycerol, 20 mM K-HEPES pH 7.4, 60 mM KCl, 0.03% (w/v) CHAPS, 3 mM $MgCl_2$, 2 mM DTT, and heavy buffer containing 40% (v/v) glycerol, 0.002% (v/v) glutaraldehyde (Sigma), 20 mM K-HEPES pH 7.4, 60 mM KCl, 0.03% (w/v) CHAPS, 3 mM $MgCl_2$, 2 mM DTT. The centrifugation was performed using an SW60 Ti rotor (Beckman Coulter) at 32,000 rpm at 4 °C for 14 hours. Subsequently, peak fractions were pooled and the cross-linking reactions were quenched with 50 mM Tris-HCl pH7.0. The homogeneity of peak fractions was assessed by negative-stain electron microscopy. Fractions of interest were concentrated to about 2.4 mg/ml and dialyzed overnight against a

buffer containing 20 mM K-HEPES pH 7.4, 60 mM KCl, 0.8% (v/v) glycerol, 1 mM tris (2-carboxyethyl) phosphine (TCEP), followed by cryo-EM grid preparation.

For negative-stain EM, 5 μl of freshly purified protein sample was applied onto a glow-discharged copper grid supported by a thin layer of carbon film for 1 min before negative staining by 2% (w/v) uranyl formate at room temperature. The negatively stained grid was loaded onto a FEI Talos L120C microscope operated at 120 kV, equipped with a Ceta CCD camera.

For cryo-EM grid preparation, 4 μl of protein sample (about 0.9 mg/ml) was applied onto a glow-discharged holey carbon grid (Quantifoil Au, R2/2, 300 mesh). After blotting for 3 s, the grid was vitrified by plunging it into liquid ethane using a Vitrobot Mark IV (FEI) operated at 4 °C and 100% humidity.

IgG pulldown assay

Expi293F cells containing overexpressed INTAC complex were pelleted and lysed as previously described (Zheng et al., 2020). The supernatant of the cell lysate was incubated with IgG resins for 2 hours at 4 °C. The INTAC complex was immobilized on the resins by N-terminal 4×Protein A–tagged INTS1. The resins were extensively washed and resuspended in 450 μl of the binding buffer containing 30 mM K-HEPES pH7.4, 100 mM KCl, 0.1% (w/v) CHAPS, 3 mM $MgCl_2$, 8% (v/v) glycerol, 2 mM DTT. The purified Pol II or Pol II with deletion of RPB1 CTD (Pol II$^{\Delta CTD}$) expressed in Expi293F cells was subjected to removing endogenous RPAP2 by incubating with RPAP2 antibody (Abclonal) on Protein G resins. The resulting Pol II, Pol II$^{\Delta CTD}$, or their mixture with DSIF and NELF, in the presence or absence of a bubble scaffold was individually incubated with INTAC-immobilized IgG resins for 2 hours at 4 °C. The resins were extensively washed with the binding buffer, and the bound proteins were subjected to SDS-PAGE followed by Coomassie blue staining. Other IgG pulldown assays were performed in a similar approach as described above.

RNA cleavage assay

To test INTAC-mediated RNA cleavage in the context of PEC, five different single-stranded RNAs (23-nt, 38-nt, 40-nt, 46-nt and 40-nt*) were used as substrates. 23, 38, 40, 46-nt RNA: 5′-U$_{46}$UA AGG A$_{40}$AU$_{38}$ UAA GUC GUG CGU CUA$_{23}$ AUA ACC GGA GAG GGA ACC CAC U-3′ (Vos et al., 2018a) (The subscripts represent four different RNA lengths from the 3′ end). 40-nt* RNA: 5′-CAA UAA ACA AGU UAA CAA CAA CAA UUG CAU GGA ACC CAC U-3′ (Boreikaite et al., 2022). These RNAs were synthesized with a 5′ 6-FAM fluorescent label by GenScript and Bioneer. Pol II EC was first formed using 400 nM of Pol II, 200 nM of annealed DNA–RNA hybrid and 400 nM non-template DNA at 30 °C and then transferred on ice. 58 nM or 26 nM (only for 46 nt RNA) INTAC, and 400 nM of DSIF and NELF were then mixed with Pol II EC on ice. All concentrations above refer to the final

concentration used in the assay. All cleavage reactions were performed in 10 µl final volume in a buffer containing 20 mM K-HEPES pH 7.4, 60 mM KCl, 3 mM $MgCl_2$, 10% (v/v) glycerol, 20 mM EDTA, 2 mM DTT, 1 U/µl RNAsin® plus (Promega). The reactions were incubated at 30 °C for 30 min and stopped by adding 10 µl of 2×stop buffer (8 M urea, 50 mM EDTA, 20% (v/v) glycerol, 1×TBE) and boiling at 70 °C for 3 minutes. 4 µl of each reaction was applied to a denaturing gel (8 M urea, 1×TBE, 20% Bis-Tris acrylamide 19:1 gel). The gel was run in 0.5×TBE buffer at 550 V for 100 min. Products were visualized using the 6-FAM label and a Typhoon 9500 FLA Imager (GE Healthcare Life Sciences). The cleavage activity of INTAC (E203Q) was determined in a similar approach as described above.

Cryo-EM data collection and image processing

Cryo-EM data were collected on a Titan Krios electron microscope (FEI) operated at 300 kV at the Cryo-EM platform of Fudan University, equipped with a K2 summit direct detector (Gatan) and a GIF quantum energy filter (Gatan) set to a slit width of 20 eV. Automated data acquisition was carried out with Serial EM software in the super-resolution mode (Mastronarde, 2005) at a nominal magnification 130,000×, corresponding to a calibrated pixel size of 1.054 Å, and a defocus range from −1.5 to −2.5 µm. Each image stack was dose fractionated to 32 frames with a total exposure dose of about 50 e^{-}/Å2 and exposure time of 6.72 s. The image stacks were motion-corrected and dose-weighted using MotionCorr2 (Zheng et al., 2017). The contrast transfer function (CTF) parameters were estimated by CTFFIND-4.1 from non-dose weighted micrographs. About 51,000 particles autopicked from 2000 micrographs were subjected into two-dimensional (2D) classification in RELION v3.0 (Scheres, 2012) and ab initio reconstruction by cryoSPARC v2 (Punjani et al., 2017). The 3D initial model was low-passed and used as references for subsequent particle-picking and 3D classification. The following procedures of image processing were performed using RELION for dose-weighted micrographs, 1,795,128 particles were autopicked from 19,469 micrographs for further data processing. After several rounds of 3D classification, 73,767 good particles were selected for further no-alignment 3D classification. Because of the relatively flexible organization between INTAC and PEC, the mask of INTS1-INTS6-INTS9-INTS11-PEC was applied to no-alignment 3D classification to separate the weakly associated INTAC-PEC. Finally, 41,201 particles (stably associated INTAC-PEC) were subjected to 3D-autorefinement, postprocessing, CTF refinement and Bayesian polishing, yielding a reconstruction of INTAC-PEC at 4.18 Å resolution. In order to improve the map quality for model building, focused classification and refinement were used. Afterwards, selected particles were postprocessed, CTF-refined, Bayesian polished and generated reconstructions of the INTAC at 3.72 Å (127,686 particles), INTS2-INTS7-CTD at 3.46 Å (127,686 particles), PP2A-AC at 3.81 Å (119,859 particles), INTS9-INTS11-Pol II-DSIF at 3.75 Å (67,991 particles), INTS9-INTS11-RNA at 3.80 Å (67,991 particles) and INTS11-RNA at 3.66 Å (67,991 particles). The reported resolutions

above are based on the gold-standard Fourier shell correlation (FSC) 0.143 criterion. All the visualization and evaluation of 3D density maps were performed with UCSF Chimera (Pettersen et al., 2004) or UCSF ChimeraX (Pettersen et al., 2021), and the local resolution variations were calculated using ResMap. The above procedures of data processing are summarized in Fig.S2.

Model building and structure refinement

The structural model of INTAC-PEC was built according to the 4.18 Å INTAC-PEC cryo-EM map and corresponding focused refined maps. The structures of human INTAC (PDB: 7CUN) and PEC (PDB: 6GML) were used to guide modeling of INTAC-PEC, which were docked into the INTAC-PEC cryo-EM map by rigid body fitting using UCSF Chimera (Pettersen et al., 2004) and were manually adjusted using COOT (Emsley and Cowtan, 2004). The models of INTS1, INTS2, INTS4, INTS8, INTS9 and INTS11 were further optimized in the guidance of the protein structures predicted by AlphaFold (Jumper et al., 2021). To build the model of INTS11 (active conformation) and RNA (−20 to −23), the homologous structure of CPSF with RNA (PDB: 6V4X) was used as a reference according to the INTS9-INTS11-RNA map and the model of RNA (−1 to −10) was built using map INTS11-RNA.

The structural model of the INTAC-PEC complex was refined against the 4.18 Å overall map in real space with PHENIX (Adams et al., 2002) and validated through examination of Ramachandran plot statistics, a MolProbity score (Chen et al., 2010), and a EMRinger score (Barad et al., 2015). The statistics of the map reconstruction and model refinement are summarized in Table S1. Each focused refined maps were used to create the composite map using UCSF ChimeraX (Pettersen et al., 2021). The composite map was used in Fig. 1A and Video S1. Map and model representations in the figures and videos were prepared by PyMOL and UCSF ChimeraX (Pettersen et al., 2021).

REFERENCE:

Adams, P.D., Grosse-Kunstleve, R.W., Hung, L.W., Ioerger, T.R., McCoy, A.J., Moriarty, N.W., Read, R.J., Sacchettini, J.C., Sauter, N.K., and Terwilliger, T.C. (2002). PHENIX: building new software for automated crystallographic structure determination. Acta Crystallogr D Biol Crystallogr 58, 1948-1954.

Barad, B.A., Echols, N., Wang, R.Y.-R., Cheng, Y., Dimaio, F., Adams, P.D., and Fraser, J.S. (2015). EMRinger: side chain–directed model and map validation for 3D cryo-electron microscopy. Nature Methods 12, 943-946.

Boreikaite, V., Elliott, T.S., Chin, J.W., and Passmore, L.A. (2022). RBBP6 activates the pre-mRNA 3' end processing machinery in humans. Genes Dev 36, 210-224.

Chen, V.B., Arendall, W.B., 3rd, Headd, J.J., Keedy, D.A., Immormino, R.M., Kapral, G.J., Murray, L.W., Richardson, J.S., and Richardson, D.C. (2010). MolProbity: all-atom structure validation for macromolecular crystallography. Acta Crystallogr D Biol Crystallogr 66, 12-21.

Chen, X., Qi, Y., Wu, Z., Wang, X., Li, J., Zhao, D., Hou, H., Li, Y., Yu, Z., Liu, W., *et al.* (2021). Structural insights into preinitiation complex assembly on core promoters. Science 372, eaba8490.

Emsley, P., and Cowtan, K. (2004). Coot: model-building tools for molecular graphics. Acta Crystallogr D Biol Crystallogr 60, 2126-2132.

Jumper, J., Evans, R., Pritzel, A., Green, T., Figurnov, M., Ronneberger, O., Tunyasuvunakool, K., Bates, R., Žídek, A., Potapenko, A., *et al.* (2021). Highly accurate protein structure prediction with AlphaFold. Nature 596, 583-589.

Kastner, B., Fischer, N., Golas, M.M., Sander, B., Dube, P., Boehringer, D., Hartmuth, K., Deckert, J., Hauer, F., Wolf, E., *et al.* (2008). GraFix: sample preparation for single-particle electron cryomicroscopy. Nat Methods 5, 53-55.

Mastronarde, D.N. (2005). Automated electron microscope tomography using robust prediction of specimen movements. J Struct Biol 152, 36-51.

Pettersen, E.F., Goddard, T.D., Huang, C.C., Couch, G.S., Greenblatt, D.M., Meng, E.C., and Ferrin, T.E. (2004). UCSF Chimera--a visualization system for exploratory research and analysis. J Comput Chem 25, 1605-1612.

Pettersen, E.F., Goddard, T.D., Huang, C.C., Meng, E.C., Couch, G.S., Croll, T.I., Morris, J.H., and Ferrin, T.E. (2021). UCSF ChimeraX: Structure visualization for researchers, educators, and developers. Protein Sci 30, 70-82.

Punjani, A., Rubinstein, J.L., Fleet, D.J., and Brubaker, M.A. (2017). cryoSPARC: algorithms for rapid unsupervised cryo-EM structure determination. Nat Methods 14, 290-296.

Scheres, S.H. (2012). RELION: implementation of a Bayesian approach to cryo-EM structure determination. J Struct Biol 180, 519-530.

Sun, Y., Zhang, Y., Aik, W.S., Yang, X.C., Marzluff, W.F., Walz, T., Dominski, Z., and Tong, L. (2020). Structure of an active human histone pre-mRNA 3'-end processing machinery. Science 367, 700-703.

Vos, S.M., Farnung, L., Boehning, M., Wigge, C., Linden, A., Urlaub, H., and Cramer, P. (2018a). Structure of activated transcription complex Pol II-DSIF-PAF-SPT6. Nature 560,

607-612.
Vos, S.M., Farnung, L., Urlaub, H., and Cramer, P. (2018b). Structure of paused transcription complex Pol II-DSIF-NELF. Nature 560, 601-606.
Xu, Y., Xing, Y., Chen, Y., Chao, Y., Lin, Z., Fan, E., Yu, J.W., Strack, S., Jeffrey, P.D., and Shi, Y. (2006). Structure of the protein phosphatase 2A holoenzyme. Cell 127, 1239-1251.
Zheng, H., Qi, Y., Hu, S., Cao, X., Xu, C., Yin, Z., Chen, X., Li, Y., Liu, W., Li, J*., et al.* (2020). Identification of Integrator-PP2A complex (INTAC), an RNA polymerase II phosphatase. Science 370, eabb5872.
Zheng, S.Q., Palovcak, E., Armache, J.P., Verba, K.A., Cheng, Y., and Agard, D.A. (2017). MotionCor2: anisotropic correction of beam-induced motion for improved cryo-electron microscopy. Nat Methods 14, 331-332.

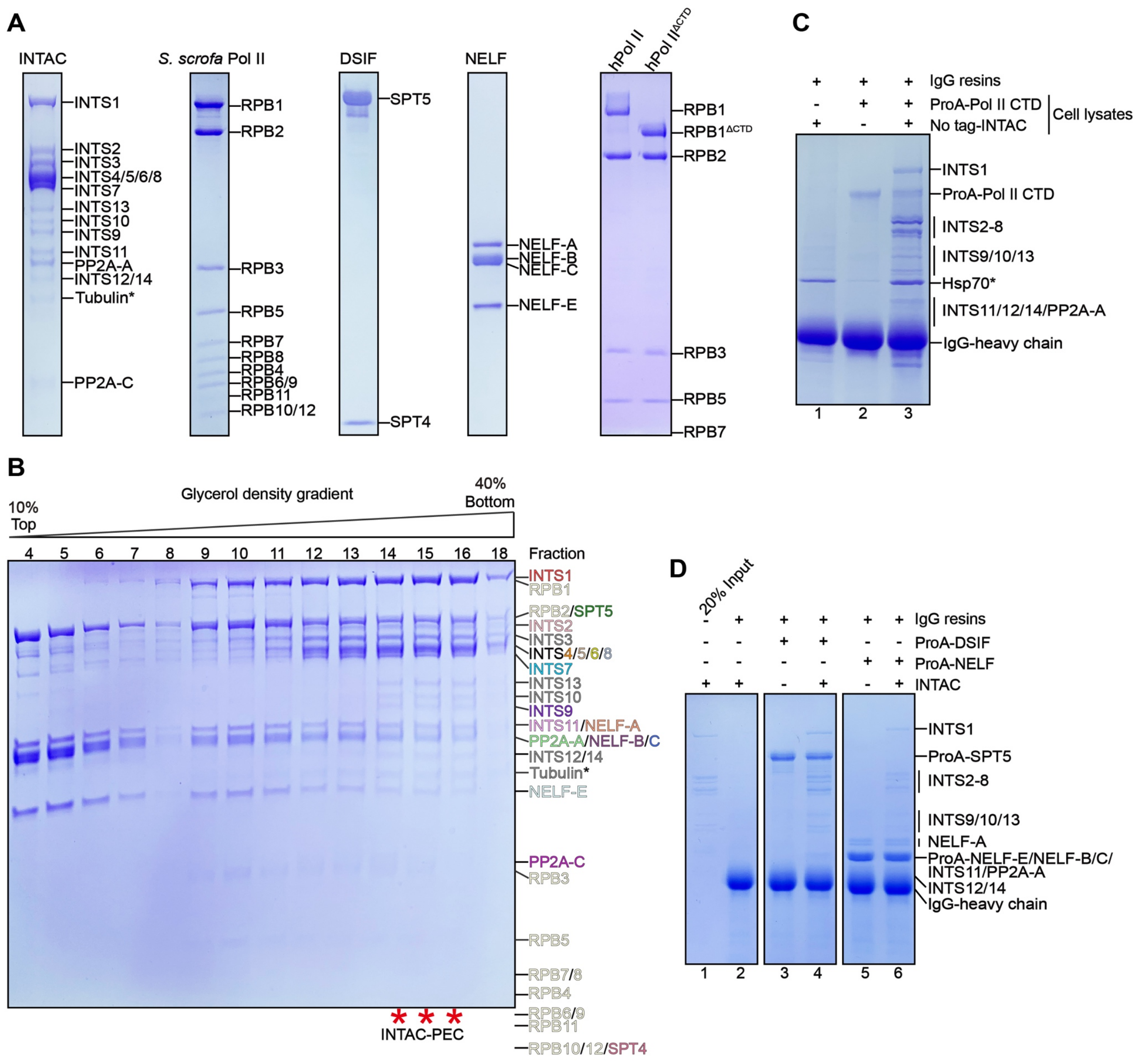

Supplementary Figure 1. Protein purification and complex assembly. (A) Protein complexes used in biochemical and structural analyses. The samples were subjected to SDS-PAGE and stained using Coomassie blue. Pol II represents RNA polymerase II purified from *S. scrofa* and hPol II represents the Pol II overexpressed and purified from Expi293F cells. hPol II$^{\Delta CTD}$ represents hPol II containing truncation of RPB1 CTD (residues 1593−1970). To better reveal phosphorylated RPB1 of hPol II and CTD truncation of RPB1, the lower bands of Pol II subunits were omitted. (B) Glycerol density gradient of the mixture of INTAC and PEC. Peak fractions of the assembled INTAC-PEC complex are indicated with red stars below. (C and D) IgG pulldown assay using protein A (ProA)–tagged CTD (C) and DSIF and NELF (D) to test their binding of INTAC. Contaminating proteins (Hsp70 and Tubulin) are indicated with black stars.

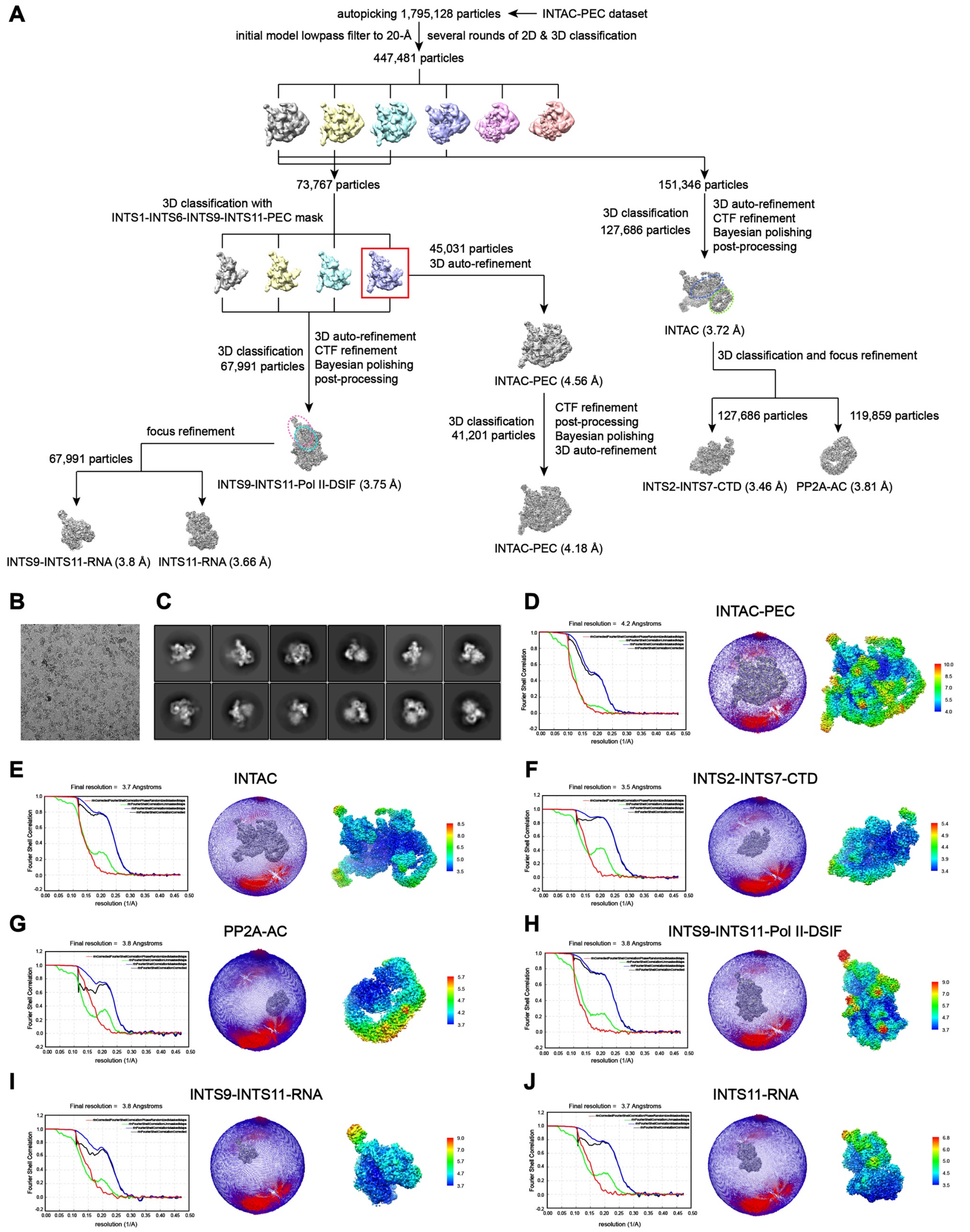

Supplementary Figure 2. Data processing. (A) Flow-charts of the cryo-EM image processing and 3D reconstructions. (B and C) Representative cryo-EM raw micrograph (B) and 2D classification (C) of INTAC-PEC. (D–J) The GSFSC curves, angular distribution plots, and local resolution estimations of the cryo-EM maps of global refinement (D) and focused refinements (E–J).

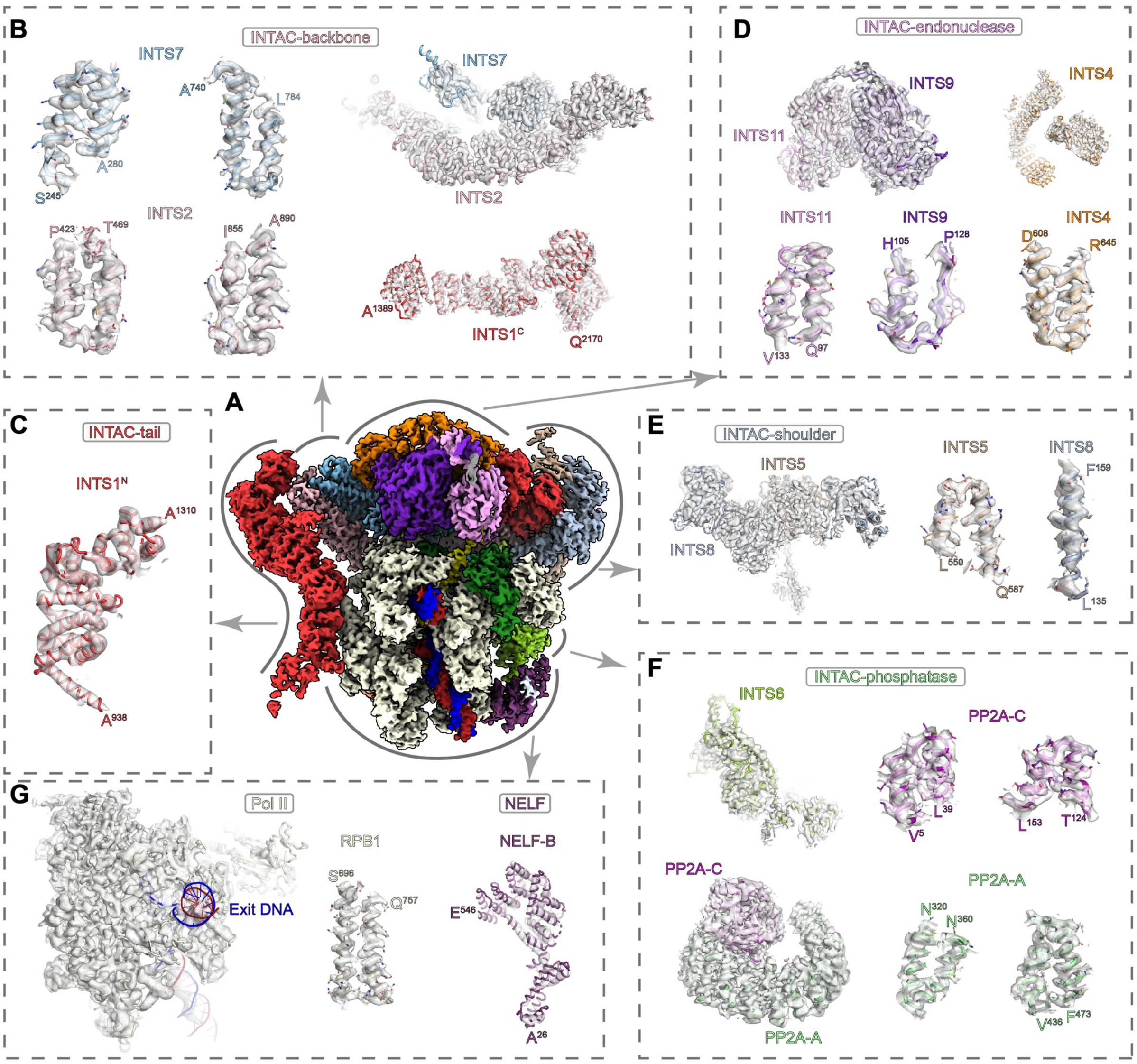

Supplementary Figure 3. Cryo-EM maps and structural models. (A) Composite cryo-EM map of INTAC-PEC as shown in Fig. 1A. The positions of each submodule shown in (B)–(G) are indicated on the overall map. (B–G) Cryo-EM density for each submodule, and close-up views of representative structural models with the corresponding cryo-EM maps shown in surface. Proteins are shown in ribbon and sticks (side chains). Most of the side chains fit into the cryo-EM map, indicating the model was built correctly.

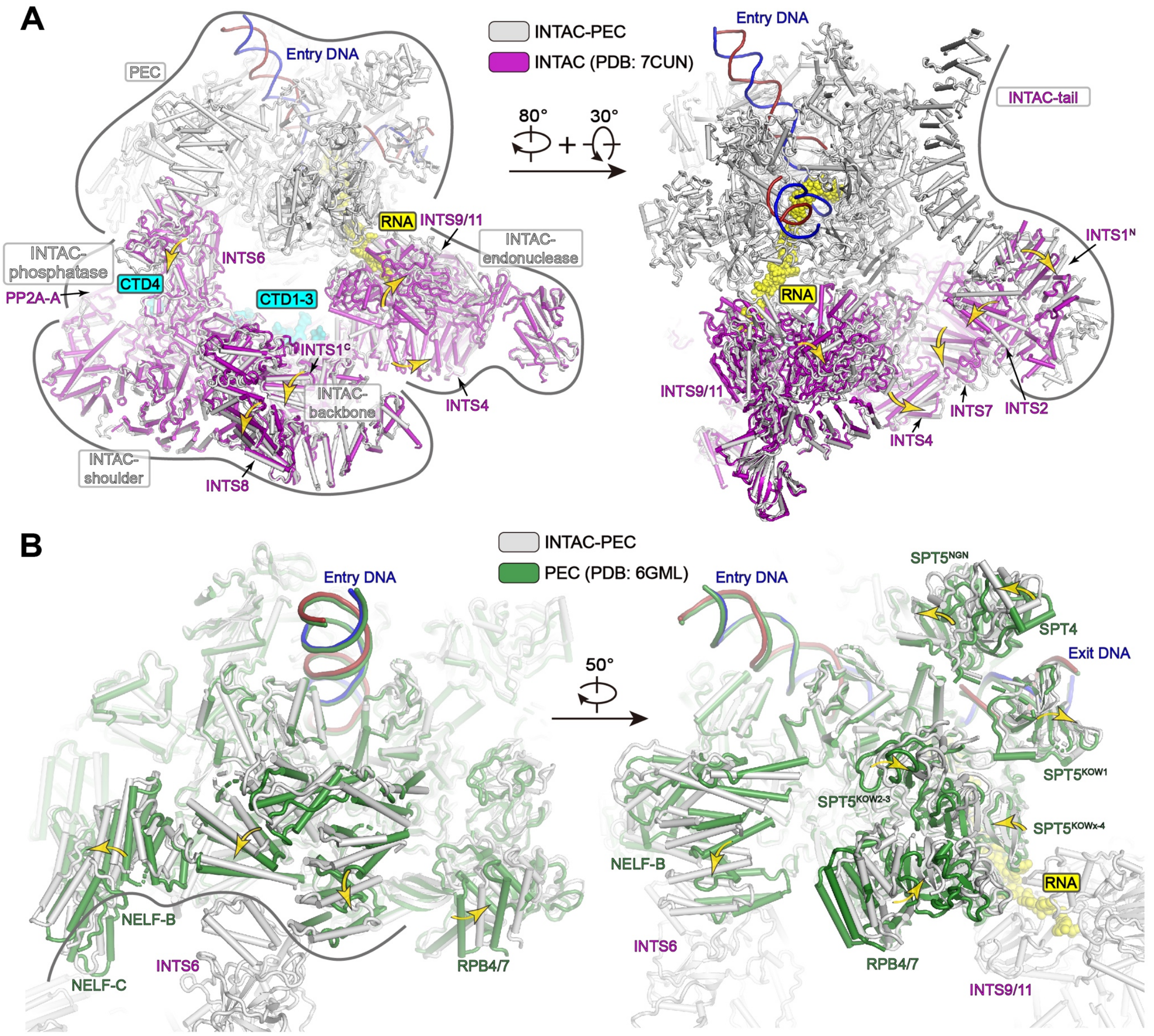

Supplementary Figure 4. Conformational changes of INTAC and PEC upon formation of INTAC-PEC. Structural comparisons of INTAC-PEC with INTAC (PDB: 7CUN) (Zheng et al., 2020) (A) and PEC (PDB: 6GML) (Vos et al., 2018b) (B). For clarity, INTAC-PEC is colored in grey and other complexes are colored as indicated. Structural differences are highlighted and modular displacements are indicated with yellow arrows. Two different views are shown for each comparison.

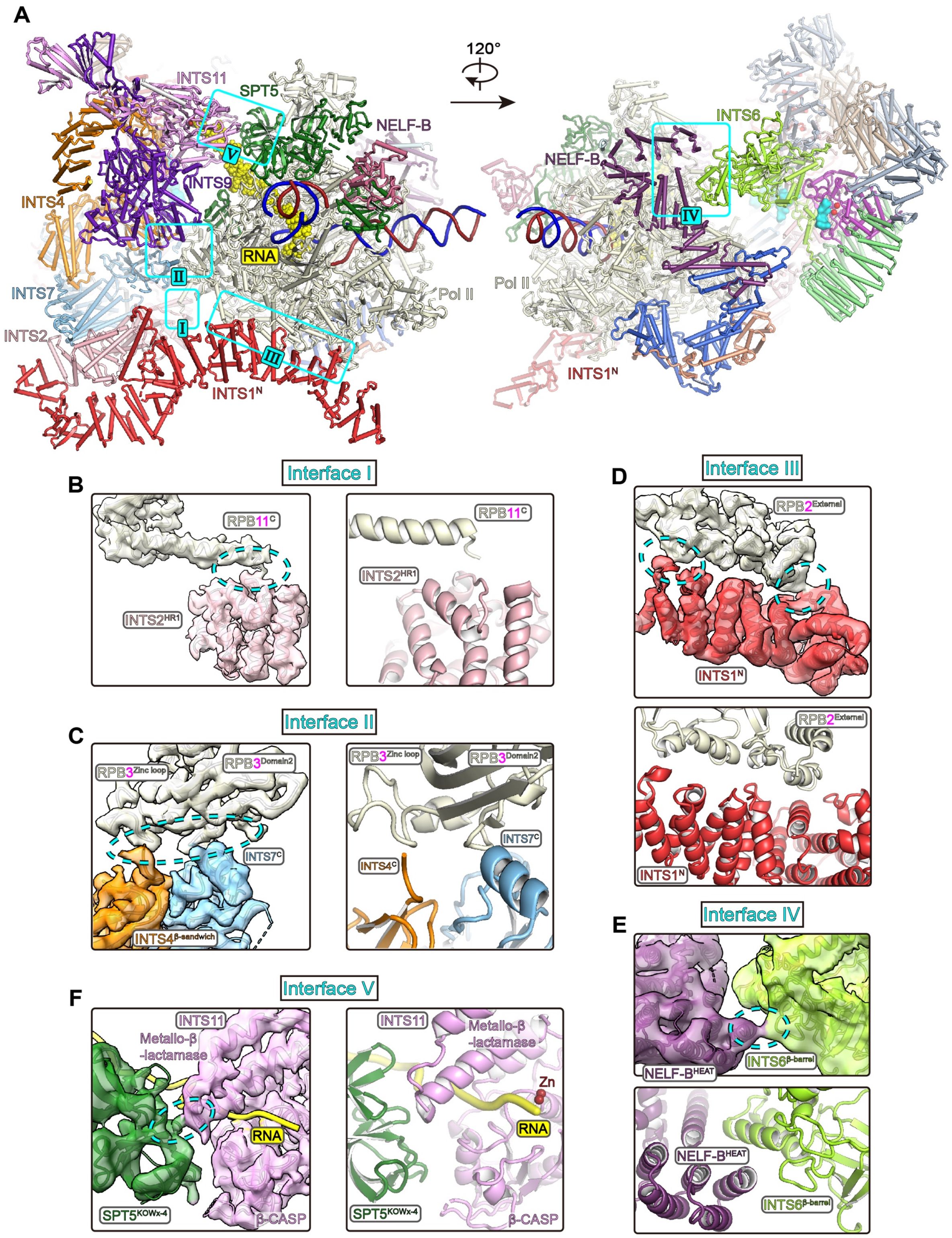

Supplementary Figure 5. Interfaces between INTAC and PEC. (A) Overall structure of INTAC-PEC with the five inter-complex contacts highlighted. (B–F) Interface-I to -V are shown with structural models (right or bottom panels) and structural models covered by transparent cryo-EM maps (left or upper panels) as shown in Fig. 1C–G. The two panels are shown in similar orientation. Contacts are highlighted with cyan dashed circles. Interactions between INTAC and Pol II CTD are omitted here and shown in Fig. S6.

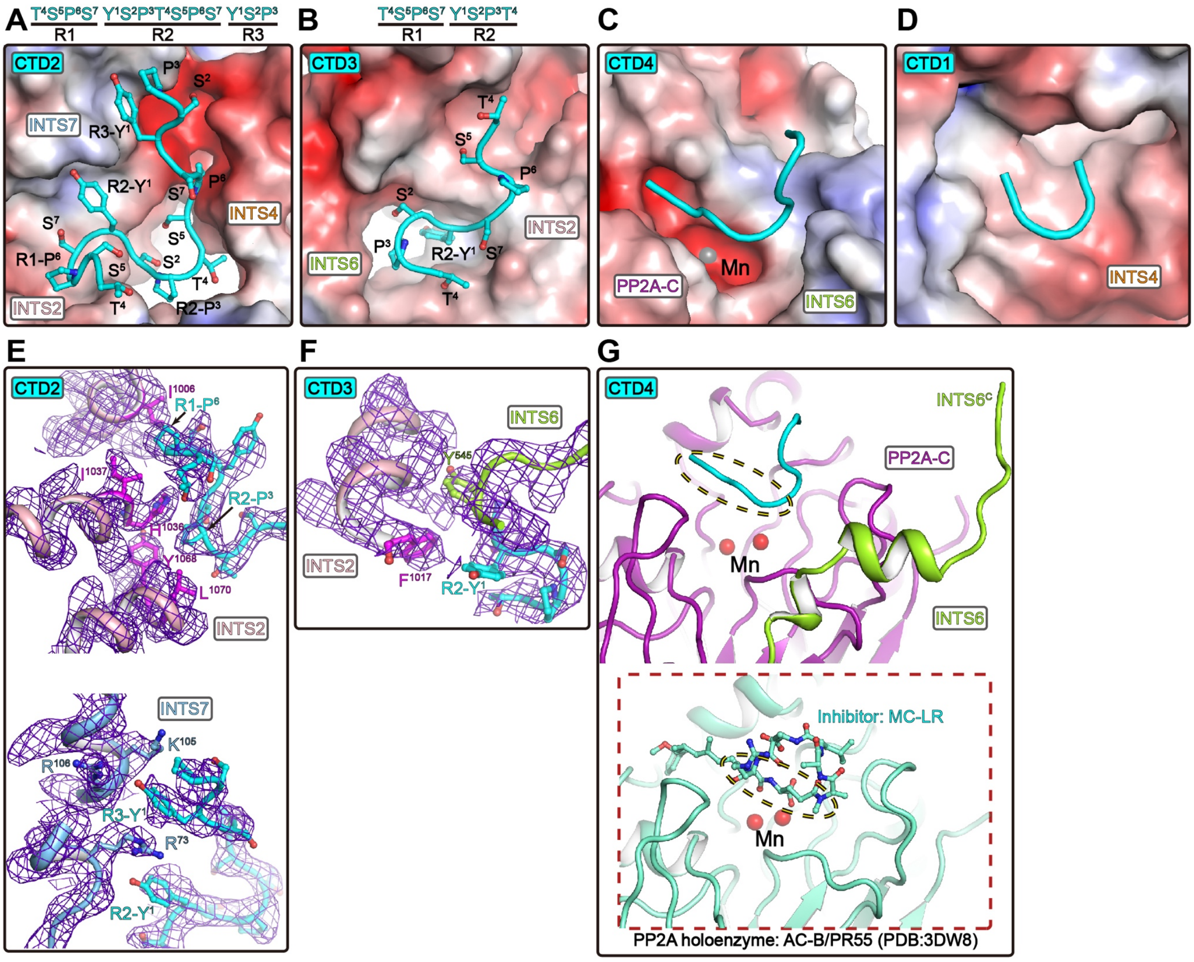

Supplementary Figure 6. **Binding of Pol II CTD to INTAC backbone and PP2A-C.** (A–D) Interactions between INTAC and four CTD segments. Electrostatic potential surface of INTAC is shown. CTD2 and CTD3 are shown in sticks with heptad peptide indicated. As shown in Fig. 2A, the cryo-EM maps did not support unambiguous assignment of CTD1 and CTD4. The two segments are shown in cartoon. R1, R2 and R3 represent the repeat number of heptad peptide. (E and F) Cryo-EM maps around CTD2 and CTD3 segments. Cryo-EM maps are shown in purple meshes. Residues that potentially involved in recognition of CTD are shown in sticks. (G) Comparison of CTD4 and MC-LR (PP2A inhibitor) in INTAC-PEC and PP2A holoenzyme (Xu et al., 2006) structures. The two PP2A-C structures are shown in a similar orientation.

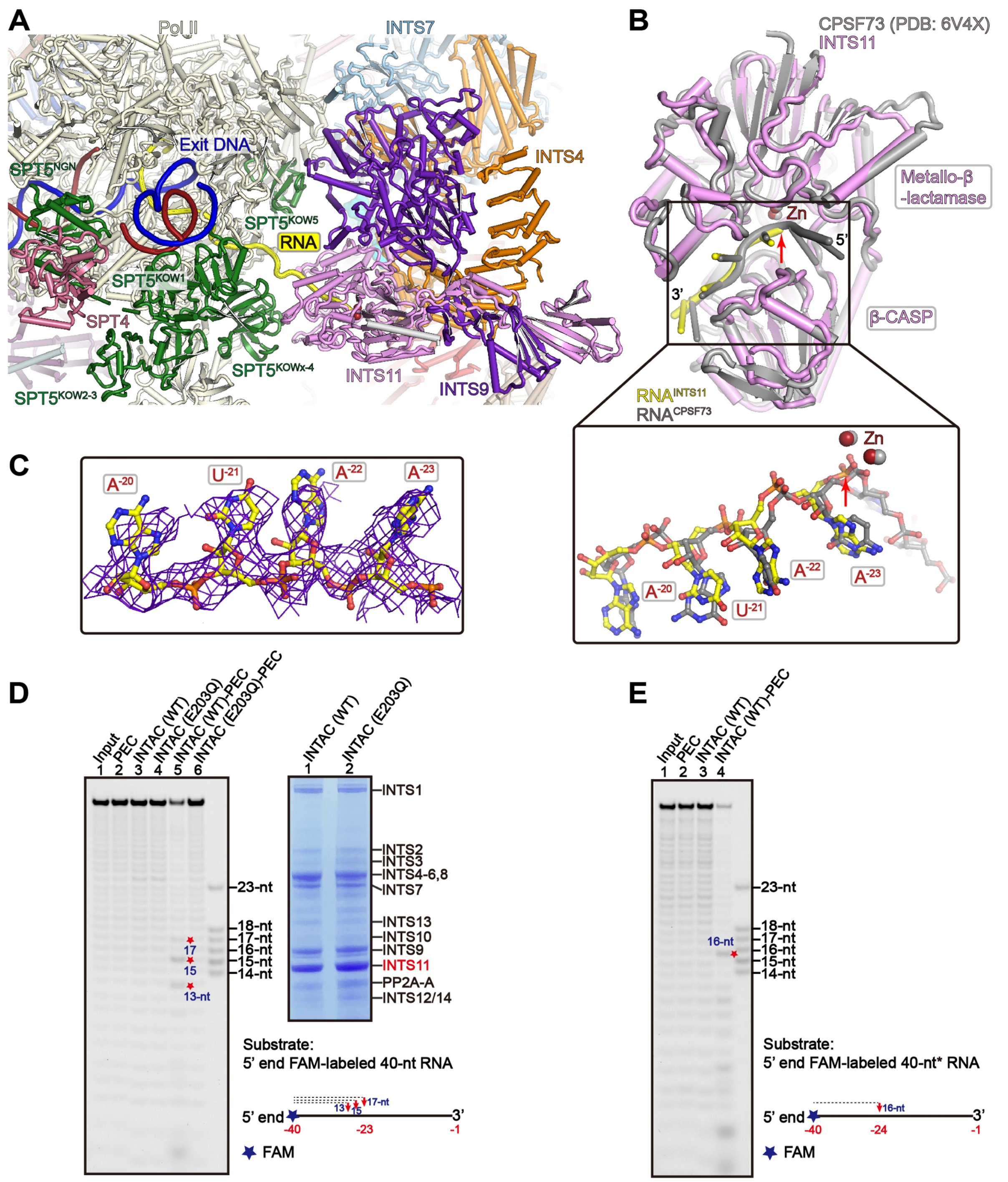

Supplementary Figure 7. Binding of RNA within the RNA entry tunnel of INTS11. (A) Contacts between PEC and endonuclease module of INTAC. (B) Structural comparison of the RNA-bound INTS11 in INTAC-PEC and the RNA-bound CPSF73 in HCC (PDB: 6V4X) (Sun et al., 2020). Note that RNA was not cleaved in HCC complex. (C) Cryo-EM map of the RNA within INTS11 is shown in mesh and the RNA is shown in sticks. (D) The patrial RNA cleavage assay of INTAC (WT) and INTAC (E203Q) using a 40-nt RNA as a substrate. INTAC (WT): wild type INTAC, INTAC (E203Q): INTAC with INTS11-E203Q mutation. Note that only FAM-labeled 5' end-containing products could be visualized. Representative RNA cleavage products are highlighted in red stars. The purified INTAC (WT) and INTAC (E203Q) were

subjected to SDS-PAGE. (E) The patrial RNA cleavage assay of INTAC (WT) using a different RNA substrate (40-nt*). Representative RNA cleavage products are highlighted in red stars.

Table S1. Cryo-EM data collection, refinement and validation statistics

	#1 INTAC-PEC (EMDB-33741) (PDB 7YCX)	#2 INTS2-INTS7-CTD	#3 INTS9-INTS11-RNA
**Data collection and processing**			
Magnification	130, 000×	130, 000×	130, 000×
Voltage (kV)	300	300	300
Electron exposure (e^{-}/Å^2)	50	50	50
Defocus range (μm)	−1.5 to −2.5	−1.5 to −2.5	−1.5 to −2.5
Pixel size (Å)	1.054	1.054	1.054
Symmetry imposed	C1	C1	C1
Initial particle images (no.)	1,795,128	1,795,128	1,795,128
Final particle images (no.)	41,201	127,686	67,991
Map resolution (Å)	4.2	3.5	3.8
FSC threshold	0.143	0.143	0.143
Map resolution range (Å)	4.0−10.0	3.4−5.4	3.7−9.0
**Refinement**			
Initial model used (PDB code)	7CUN, 6GML		
Model resolution (Å)	4.2		
FSC threshold	0.5		
Map sharpening *B* factor (Å^2)	−90	−92	−105
Model composition			
Non-hydrogen atoms	113,089		
Protein residues	14,682		
Nucleotide residues	104		
Ligands	MN: 2, MG: 1, ZN: 10		
B factors (Å^2)			
Protein	96.79		
Nucleotide	232.35		
Ligand	55.21		
R.m.s. deviations			
Bond lengths (Å)	0.005		
Bond angles (°)	0.998		
Validation			
MolProbity score	1.96		
Clashscore	11.38		
Poor rotamers (%)	0.18		
Ramachandran plot			
Favored (%)	94.32		
Allowed (%)	5.53		
Disallowed (%)	0.15		

Supplementary Video 1

Composite cryo-EM map and structural model of INTAC-PEC.

Supplementary Video 2

The CTD-binding path on INTAC. Cryo-EM map and structural model of CTD-binding path and four putative CTD fragments are shown. Pol II is shown to indicate its relative position to the CTD-binding path. The last modeled residue of RPB1 (P1487) is indicated with a red ball.

Supplementary Video 3

Cryo-EM map and structural model of PEC and endonuclease module of INTAC. The RNA is colored in yellow.

Supplementary Video 4

Binding of RNA-bound PEC to INTAC leads to activation of INTS11. Non-related modules were omitted for simplicity.
